# Impact of Gamma COVID-19 variant on the prognosis of hospitalized pregnant and postpartum women with cardiovascular disease

**DOI:** 10.1016/j.clinsp.2024.100454

**Published:** 2024-08-08

**Authors:** Carolina Burgarelli Testa, Luciana Graziela de Godoi, Nátaly Adriana Jiménez Monroy, Maria Rita de Figueiredo Lemos Bortolotto, Agatha Sacramento Rodrigues, Rossana Pulcineli Vieira Francisco

**Affiliations:** a Divisão de Clínica Obstétrica, Hospital das Clínicas, Faculdade de Medicina, Universidade de São Paulo (HCFMUSP), São Paulo, SP, Brazil; b DaSLab (Data Science Lab), Department of Statistics, Universidade Federal do Espírito Santo, Vitória, ES, Brazil; c Disciplina de Obstetrícia, Departamento de Obstetrícia e Ginecologia, Faculdade de Medicina, Universidade de São Paulo (FMUSP), São Paulo, SP, Brazil

**Keywords:** COVID-19 Gamma Variant, Pregnancy, Cardiovascular Diseases

## Abstract

*Objective*: The aim of the study was to assess the impact of the Gamma coronavirus disease 2019 (COVID-19) variant on pregnant and postpartum women with Cardiovascular Disease (CVD).

*Methods*: The Influenza Epidemiological Surveillance System database (SIVEP-Gripe), a compulsory notification system for cases of Severe Acute Respiratory Syndrome (SARS), was investigated for notified cases of pregnant and postpartum women with reported CVD and SARS due to COVID-19 between February 16, 2020 and May 1, 2021 (when vaccination began), was investigated. In this retrospective cohort, two groups were formed based on symptom onset date, according to the predominance of the variants: original (group 2020) and Gamma (group 2021). Cases with missing information on the presence or absence of CVD were excluded. The comparative analysis was controlled for confounding variables.

*Results*: Among 703 COVID-19 cases notified with CVD (406 patients in 2020 and 297 patients in 2021), compared to 2020, cases in 2021 had more respiratory symptoms (90.6 % vs. 80.1 %, *p <* 0.001), greater ventilatory support need (75.3 % vs. 53.9 %, *p <* 0.001), more ICU admission (46.6 % vs. 34.3 %, p = 0.002), longer duration (20.59 ± 14.47 vs. 16.52 ± 12.98 days, *p <* 0.001), higher mortality (25.6 % vs. 15.5 %, *p <* 0.001), with more than two-times mortality likelihood in the third trimester (adjusted OR = 2.41, 95 % CI 1.50–3.88, *p <* 0.001) or puerperium periods (adjusted_OR = 2.15, 95 % CI 1.34–3.44, p = 0.001).

*Conclusions*: In Brazil, pregnant and postpartum women with CVDs in the Gamma variant phase have higher morbidity and mortality than those affected by the original variant of Coronavirus-19.

## Highlights

• Respiratory symptoms common in the 2021 Gamma outbreak in pregnant women.
• CVD caused poor outcomes in pregnant/postpartum women with COVID-19.
• Gamma COVID-19 was most lethal for pregnant/postpartum women with CVD in Brazil.
• This study underscores the Gamma variant's significant impact on maternal outcomes.

## Introduction

The Coronavirus Disease 2019 (COVID-19) pandemic witnessed a significant increase in maternal deaths.^1^^,^^2^ Accordingly, pregnant and postpartum women with Cardiovascular Diseases (CVDs) require attention in this context, as they have a higher maternal mortality rate than the general population,^3^, ^4^, ^5^, ^6^ and CVD is an isolated risk marker for COVID-19 complications in the general population.^7^

Since the start of the pandemic, COVID-19 infection surmounted 704,000 deaths in Brazil,^8^^,^^9^ including at least 2,065 maternal deaths.^2^^,^^10^, ^11^, ^12^ Temporal distribution of deaths reveals the first epidemic wave, which presented a plateau between May and September 2020, and the second wave, with a rise beginning in March 2021, a peak in April, and a decrease in June 2021.^8^ The authors observed a significantly higher number of deaths in 2021, with 1,518 maternal deaths (73.5 % of the total maternal deaths in 2020 and 2021).^13^

In November 2020, a Brazilian variant of SARS-CoV-2, known as P.1, also called the Gamma variant, was identified.^14^ The mutations identified in this variant were responsible for higher virulence and greater resistance to antibodies.^15^ Due to its greater transmissibility, the Gamma variant displayed a rapid expansion, and as of January 2021 was the most prevalent in the Brazilian population.^11^^,^^12^

Given the possible difference between the pathogenicity of the variants, it is important to assess whether the morbidity and mortality associated with the Gamma variant in the group of pregnant and postpartum women with CVD had an impact on the primary clinical characteristics of symptoms, length of hospital stay, ICU admission, need for ventilatory support and death. The aim of the study was to assess the impact of the Gamma variant on pregnant and postpartum women with CVD.

## Methods

During the COVID-19 pandemic, the cases of hospitalized patients with Severe Acute Respiratory Syndrome (SARS) were compulsorily notified to the Brazilian Ministry of Health, through the Influenza Epidemiological Surveillance System (SIVEP-Gripe).

Through the SUS (*Sistema Único de Saúde*) open data system, access to anonymized data from the SIVEP (Sistema de Informação de Vigilância Epidemiológica da Gripe)-influenza database is allowed, without individual identification of the patient.^16^^,^^17^ Ethical review and approval were waived for this study due to an open-base study, according to Brazilian regulations.

A positive case of SARS is defined as an individual who has flu-like syndrome, with at least two of the following signs and symptoms: fever, chills, sore throat, headache, cough, runny nose, olfactory disorders or taste disorders), who present: dyspnea/respiratory distress or persistent pressure or pain in the chest or O_2_ saturation lower than 95 % in room air or bluish coloration (cyanosis) of the lips or face.^18^

On May 5, 2021, the authors searched the SIVEP-influenza database for cases reported in the period between the start of the COVID-19 epidemic in Brazil (from February 16, 2020, the eighth epidemiological week of 2020) and immediately before the start of vaccination of pregnant and postpartum women (until May 1, 2021, the 17^th^ epidemiological week of 2021). The study design was conducted in accordance with STROBE statement guidelines.

In this observational retrospective cohort, SARS cases with a diagnosis of COVID-19 confirmed by the Ministry of Health criteria (91.9 % with laboratory diagnosis) who were hospitalized were selected. Subsequently, the authors selected pregnant and postpartum women aged between 10 and 55 years, with final notification and who had information on the presence of CVD. Cases with missing information on the presence or absence of CVD were excluded.

Two groups were formed according to the date of onset of symptoms, according to the predominance of the original variants: 2020 (between February 16, 2020, and December 31, 2020) and Gamma variant, 2021 (from January 1, 2020 to May 1, 2021).

The variables evaluated were the: date of onset of symptoms, age, race, education, time of pregnancy, risk factors, and declared comorbidities (hemopathy, liver disease, asthma, diabetes, neuropathy, lung disease, immunodepression, nephropathy and obesity), symptoms, ICU admission, ventilatory support, orotracheal intubation and outcome.

The analyses were performed using the statistical software *R*,^19^ with qualitative variables presented as absolute frequency (n) and percentage (%) and quantitative variables presented as mean ± standard deviation. The comparison between the groups for categorical variables was performed using the chi-square test with Yates' continuity correction, and, when necessary, Fisher's exact test. In addition, Odds Ratios (OR), with 95 % Confidence Interval (95 % CI) were also calculated. For continuous variables, the comparison between the independent groups was analyzed using the Student *t*-test or Wilcoxon rank sum test with continuity correction, when appropriate. The results were considered statistically significant when *p <* 0.05.

Considering that this is a non-experimental study, the groups of 2020 and 2021 were balanced with respect to age, ethnic group, obesity, gestational moment, and the presence of diabetes (potential confounding variables in the study) in order to control selection biases. Propensity Score Matching (PSM) was used for estimating and assessing the balancing weights of the observations to make two balanced groups through the Inverse Probability of Treatment Weighting Method (IPTW). Logistic regression was the method used to create the propensity score weights and the Average Treatment Effect (ATE) was estimated for treatment effects based on IPTW. Analyses related to the comparison of symptoms and outcomes in the groups with and without CVD were redone considering the PSM weights. PSM was carried out with the R Weightlt package.^20^^,^^21^

## Results

**Fig. 1:**
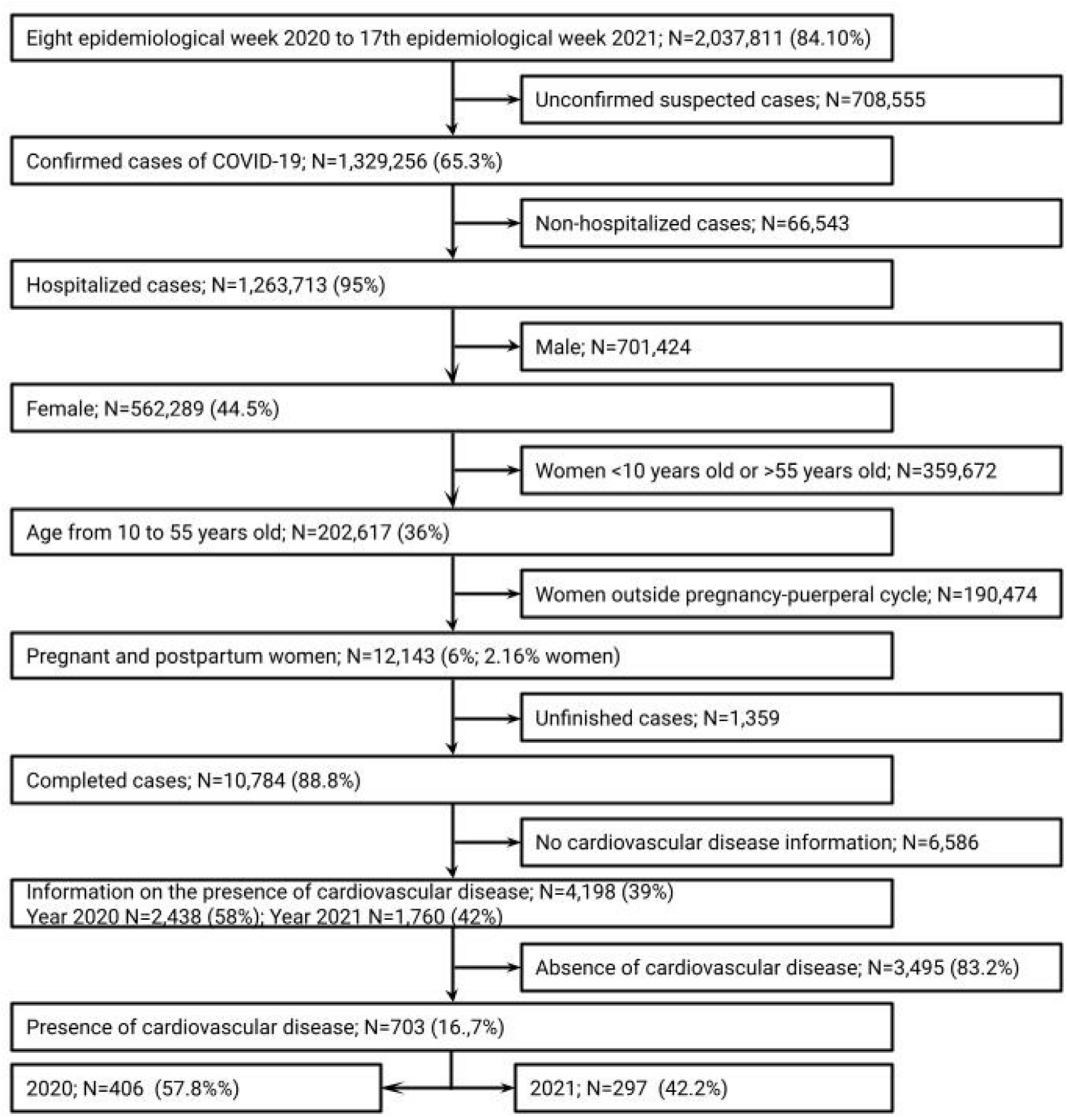
Case selection.

The cases of COVID-19 reported in SIVEP-Influenza between the 8^th^ epidemiological week of 2020 (which corresponds to the beginning of the COVID-19 epidemic in Brazil) and the 17^th^ epidemiological week of 2021 (immediately before the start of vaccination of pregnant and postpartum women) were evaluated. The selection of cases is described in Figure 1.

The present study group was composed of 703 patients with referred CVD, 406 patients in 2020 and 297 patients in 2021. Still regarding the age variable, the youngest woman with CVD was 15 years old and 41 women with CVD were at least 50 years old, 9 of them were 55 years old.

Table 1 presents the epidemiological data. There was no difference in age and disease profile associated with CVD between 2020 and 2021 years, except for obesity, which was more frequent during the period of predominance of the Gamma variant. Brown women were more frequently affected in 2020, while white women were more affected in 2021, indicating that, among those with heart disease, there was a change in the profile of those affected by COVID-19 with respect to race.

**Table 1:** Demographic and clinical characteristics of pregnant and postpartum women with Cardiovascular Disease (CVD) hospitalized with confirmed COVID-19 in the years 2020 and 2021 – Brazil, 02/16/2020 to 05/01/2021.

Patient Characteristics	2020	2021	2021 versus 2020	
Pregnant and Puerperal Women with CVD	n = 406 (16.7 %)	n = 297 (16.9 %)	Odds Ratio	p
**Ethnic category**	**n (%)**	**n (%)**		
Yellow	1 (0.3)	1 (0.4)		0.020
White	124 (36.6)	125 (49.0)		
Indigenous	1 (0.3)	1 (0.4)		
Brown	181 (53.4)	108 (42.4)		
Black	32 (9.4)	20 (7.8)		
**Schooling**	**n (%)**	**n (%)**		
None	1 (0.6)	1 (0.7)		0.477
1^st^–5^th^ grade	20 (11.0)	19 (13.9)		
6^th^–9^th^ grade	30 (16.6)	29 (21.2)		
High school	95 (52.5)	70 (51.1)		
Higher education	35 (19.3)	18 (13.1)		
**Age (years)**	**Mean ± SD**	**Mean ± SD**		
	33.96 ± 8.11	34.96 ± 8.49		0.117
**Age group**	**n (%)**	**n (%)**		
< 20 years old	10 (2.5)	2 (0.7)		0.128
20–34 years old	208 (51.2)	145 (48.8)		
≥ 35 years old	188 (46.3)	150 (50.5)		
**Gestational period**	**n (%)**	**n (%)**		
1^st^ trimester	20 (4.9)	27 (9.1)		0.04
2^nd^ trimester	74 (18.2)	70 (23.6)		
3^rd^ trimester	173 (42.6)	111 (37.4)		
Unknown GA	18 (4.4)	16 (5.4)		
Puerperium	121 (29.8)	73 (24.6)		
**Associated diseases**	**n (%)**	**n (%)**		
Hematologic	8 (2.7)	2 (0.9)	0.32 (0.03‒1.62)	0.199
Diabetes	101 (32.5)	81 (33.3)	1.04 (0.73‒1.49)	0.903
Obesity	64 (21.6)	70 (29.4)	1.51 (1.02‒2.24)	0.049
Asthma	26 (8.9)	18 (8.0)	0.89 (0.48‒1.67)	0.849
Liver diseases	3 (1.0)	2 (0.9)	0.85 (0.07‒7.49)	0.999
Neuropathies	5 (1.7)	7 (3.1)	1.81 (0.57‒5.79)	0.469
Lung diseases	10 (3.4)	3 (1.3)	0.38 (0.10‒1.41)	0.224
Immunodeficiencies	10 (3.5)	7 (3.1)	0.90 (0.34‒2.41)	0.999
Kidney disease	11 (3.8)	11 (4.9)	1.29 (0.55‒3.03)	0.715
At least one associated disease	172 (51.7)	147 (56.5)	1.22 (0.88‒1.69)	0.271
**Number of associated diseases**	**n (%)**	**n (%)**		
0	161 (48.3)	113 (43.5)		0.584
1	121 (36.3)	108 (41.5)		
2	41 (12.3)	30 (11.5)		
>2	10 (3.0)	9 (3.5)		

Regarding the distribution of patients according to the trimester of pregnancy at the time of admission, the most frequent gestational period at the evaluated times was the third trimester. Nevertheless, in 2021, there was a reduction in the percentage of patients who were in the third trimester and an increase in the proportion of cases hospitalized in the first and second trimesters of pregnancy (Table 1).

In particular, symptoms of dyspnea, respiratory and oxygen saturation lower than 95 % were more prevalent in 2021 (Table 2). Regarding the number of respiratory symptoms, in 2021, at least 3 respiratory symptoms were reported in 48.6 % of the cases of COVID-19 among pregnant and postpartum women with CVD. This was a much higher percentage than in 2020, at 31.9 % of cases.

**Table 2:** Symptoms of COVID-19 in pregnant and postpartum women with CVD hospitalized with confirmed COVID-19 in the years 2020 and 2021 – Brazil, 02/16/2020 to 05/01/2021.

	2020	2021	2021 versus 2020		Weighted Propensity Score Matching	
Symptoms	n (%)	n (%)	OR	p	OR	p
Fever	228 (60.8)	178 (66.9)	1.30 (0.94‒1.81)	0.134	1.25 (1.00‒1.58)	0.062
Cough	282 (74.0)	219 (78.8)	1.30 (0.90‒1.88)	0.186	1.19 (0.92‒1.53)	0.210
Sore throat	86 (25.4)	58 (25.1)	0.98 (0.67‒1.44)	0.999	0.95 (0.73‒1.25)	0.787
Smell	42 (23.1)	66 (28.2)	1.31 (0.84‒2.05)	0.284	1.26 (0.92‒1.73)	0.180
Taste	46 (25.3)	61 (25.6)	1.02 (0.65‒1.59)	0.999	1.00 (0.73‒1.37)	0.999
Diarrhea	41 (12.3)	33 (14.2)	1.18 (0.72‒1.93)	0.592	1.02 (0.72‒1.45)	0.968
Vomiting	39 (11.7)	30 (13.2)	1.14 (0.68‒1.89)	0.713	0.98 (0.69‒1.40)	0.991
Abdominal pain	21 (11.9)	21 (9.2)	0.75 (0.40‒1.43)	0.481	0.77 (0.49‒1.20)	0.302
Fatigue	53 (28.8)	77 (32.1)	1.17 (0.77‒1.77)	0.536	1.15 (0.85‒1.54)	0.408
Dyspnea	255 (67.6)	222 (80.4)	1.97 (1.36‒2.84)	<0.001	1.93 (1.49‒2.48)	<0.001
Respiratory distress	204 (55.9)	166 (65.6)	1.51 (1.08‒2.10)	0.019	1.46 (1.16‒1.84)	0.002
Saturation < 95 %	181 (50.3)	184 (69.2)	2.22 (1.59‒3.09)	<0.001	1.91 (1.52‒2.40)	<0.001
Symptoms, ≥ 1	387 (95.6)	286 (97.9)	2.22 (0.87‒5.66)	0.135	2.02 (1.07‒3.81)	0.039
Respiratory symptom, ≥ 1	314 (80.1)	259 (90.6)	2.38 (1.49‒3.80)	<0.001	2.19 (1.59‒3.01)	<0.001

In 2021 there was an increase in the frequency of patients admitted to the ICU, but the mean length of stay did not change. When assessing the need for ICU at the time of pregnancy or puerperium, the authors observed that the risk of admission in the 3^rd^ trimester was 2.4-fold higher in 2021 and similar in the other stages of pregnancy. The need for ventilatory support was more frequent in 2021. The risk of orotracheal intubation was similar in the 1^st^ and 2^nd^ trimesters and in the puerperium, and almost quadrupled for 3^rd^ trimester pregnant women in 2021 (Table 3).

**Table 3:** Outcome comparison between 2020 and 2021 of pregnant and postpartum women with cardiovascular disease hospitalized with confirmed COVID-19, Brazil, 02/16/2020 to 05/01/2021.

	2020	2021	2021 versus 2020		Weighted Propensity Score Matching	
ICU	n (%)	n (%)	OR	p	OR	p
	131 (34.3)	132 (46.6)	1.67 (1.22‒2.29)	0.002	1.58 (1.27‒1.97)	<0.001
**ICU by pregnancy period**	**n (%)**	**n (%)**				
1^st^ trimester	8 (40.0)	8 (32.0)	0.71 (0.21‒2.41)	0.807	0.56 (0.24‒1.30)	0.256
2^nd^ trimester	28 (39.4)	32 (48.5)	1.45 (0.73‒2.85)	0.371	1.27 (0.79‒2.05)	0.389
3^rd^ trimester	43 (26.7)	52 (48.6)	2.59 (1.55‒4.34)	<0.001	2.43 (1.70‒3.49)	<0.001
Puerperium	49 (43.0)	34 (48.6)	1.25 (0.69‒2.28)	0.557	1.27 (0.84‒1.92)	0.294
**Days of ICU stay**	**Mean ± SD**	**Mean ± SD**				
Mean ± SD	13.67 ± 16.39	14.95 ± 13.10		0.219		0.809
**Ventilatory support**	**n (%)**	**n (%)**				
No	172 (46.1)	69 (24.7)		<0.001		<0.001
Yes, non-invasive	139 (37.3)	126 (45.2)				
Yes, invasive	62 (16.6)	84 (30.1)				
**Orotracheal intubation**	**n (%)**	**n (%)**				
	62 (16.6)	84 (30.1)	2.16 (1.49‒3.14)	<0.001	2.01 (1.54‒2.62)	<0.001
**Orotracheal intubation by pregnancy period**	**n (%)**	**n (%)**				
1^st^ trimester	3 (16.7)	6 (26.1)	1.74 (0.30‒12.67)	0.706	1.85 (0.65‒5.23)	0.363
2^nd^ trimester	14 (20.6)	19 (29.2)	1.59 (0.72‒3.53)	0.341	1.39 (0.80‒2.43)	0.309
3^rd^ trimester	14 (8.9)	30 (27.8)	3.96 (1.98‒7.90)	<0.001	3.79 (2.27‒6.31)	<0.001
Puerperium	28 (24.8)	24 (35.3)	1.66 (0.86‒3.19)	0.179	1.55 (0.98‒2.44)	0.077
**Outcome**	**n (%)**	**n (%)**				
Recovery	343 (84.5)	221 (74.4)	1.87 (1.29‒2.72)	0.001	1.93 (1.48‒2.52)	<0.001
Death	63 (15.5)	76 (25.6)				
**Death by pregnancy period**	**n (%)**	**n (%)**				
1^st^ trimester	3 (15.0)	5 (18.5)	1.28 (0.21‒9.42)	0.999	1.41 (0.48‒4.15)	0.729
2^nd^ trimester	15 (20.3)	17 (24.3)	1.26 (0.57‒2.77)	0.705	1.30 (0.74‒2.29)	0.437
3^rd^ trimester	18 (10.4)	24 (21.6)	2.38 (1.22‒4.62)	0.015	2.41 (1.50‒3.88)	<0.001
Puerperium	23 (19.0)	24 (32.9)	2.09 (1.07‒4.07)	0.044	2.15 (1.34‒3.44)	0.001
**Days between onset of symptoms and outcome**	**Mean ± SD**	**Mean ± SD**		**CVD**		
Any outcome	16.52 ± 12.98	20.59 ± 14.47		<0.001		<0.001
Recovery	15.94 ± 12.27	19.66 ± 14.91		0.002		0.012
Death	19.56 ± 16.03	23.24 ± 12.85		0.036		0.166

The disease was, on average, more prolonged in 2021, particularly for patients who recovered.

Patients who had the disease in 2021 also had higher mortality compared to 2020. While mortality was 15.5 % in 2020 in the group of women with CVD, in 2021 it was 25.6 %. The assessment at the time of pregnancy showed that the risk of death was similar between 2020 and 2021 for patients in the 1^st^ and 2^nd^ trimesters and higher in 2021 for patients in the 3^rd^ trimester and in the puerperium period. The risk of death was also similar among patients undergoing orotracheal intubation in the two periods evaluated. However, the mortality rate among these patients was extremely high, at 59.6 %. Table 3 demonstrates the comparison of outcomes between 2020 and 2021.

## Discussion

Patients with CVD who contracted the COVID-19 virus in 2021 were more symptomatic from a respiratory point of view, needing more noninvasive and invasive ventilatory support, and having a higher risk of ICU admission, with the risk of ICU admission for 3^rd^ trimester pregnant women being 2.4-fold higher. The disease had a longer course to outcome and mortality was higher, with more than twice the likelihood of death in the 3^rd^ trimester and puerperium period.

Several studies have evaluated clinical outcomes in obstetric populations with COVID-19 and reported pregnancy and the puerperium period as risk factors for ICU admission, with an OR of 1.5 to 6.6^22^^,^^23^ and the need for invasive ventilatory support in up to 23 % of cases.^24^

The presence of CVD has also been recognized as a negative prognostic marker for the clinical course of COVID-19 patients. In a multicenter study that evaluated 1,044 patients with congenital CVD with COVID-19 (87 % cases of laboratory confirmation of COVID-19), 51 % of women and 23 pregnant women at the time of infection, there were 24 deaths due to COVID-19 (2.3 %). Anatomical complexity was not related to prognosis, but there was a higher proportion of deaths in patients with worse functional class (p = 0.002). The overall mortality reported above is lower than that found in this study since outpatients were included. Among the 67 ICU admissions, 36 cases received invasive ventilation (53.7 %) and, among those submitted to invasive ventilation, mortality was 52.^25^ In the present study, which included only hospitalized patients, the rates of invasive ventilatory support were 16.6 % in 2020, and 30 % in 2021, lower than those found by Broberg et al.,^25^ but the mortality rate among patients undergoing OTI was comparable to that reported by the authors (59.6 %), which reaffirms the peculiarity of the population with CVD, in particular the obstetric population.

Alizadehsani et al.^26^ evaluated 660 CVD patients hospitalized in Iran between January 2020 and January 2021, and found a mortality of 15.5 %, with no difference between men and women, and a positive correlation between mortality and symptoms: loss of consciousness (*p <* 0.001), decrease in saturation < 93 % (*p <* 0.001) and need for mechanical ventilation (*p <* 0.001). Women's hospitalization was, on average, longer (7.03 ± 6.97, p = 0.004).^26^ The authors noticed that the markers found in this study that showed a positive correlation with mortality in patients with CVD were more frequent in hospitalized patients throughout 2021, more often had a drop in saturation, need for OTI, and increased length of hospital stay.

Other studies that included hospitalized CVD patients, regardless of the puerperal pregnancy cycle, found a five-fold higher mortality risk compared to individuals without CVD (OR = 4.85; 95 % CI 3.06–7.70),^27^ as well as a risk of severe disease progression (27.8 % vs. 8.8 %) and higher mortality rate (22.2 % vs. 9.8 %).^28^ In a meta-analysis of 423,117 patients, Dessie et al.^29^ found a 17.62 % mortality rate in hospitalized patients with COVID-19 (95 % CI 14.26 %–21.57 %) and the presence of CVD was a marker of negative prognosis (pOR = 1.83, 95 % CI 1.50‒2.17). Raj et al.,^30^ 2023, evaluated in-hospital mortality and complicated COVID-19 infection in adult patients with congenital heart disease by evaluating a database that includes approximately 20 % of US hospitals. Among 4,219 patients with congenital heart disease, 639 (15.1 %) patients died (OR = 1.04, 95 % CI 1.04–1.04, *p <* 0.01) and 1290 (28.6 %) presented complicated conditions (OR = 1.30, 95 % CI 1.11–1.53, *p <* 0.01). In this sample, adjusted OR (aOR) was calculated. Age (aOR = 1.03, 95 % CI 1.01‒1.05), malnutrition (aOR = 2.16, 95 % CI 1.35‒3.44) and liver disease (aOR = 5.55, 95 % CI 3.13‒9.82) were correlated with mortality.^30^

Few studies have evaluated the association between CVD and the puerperal pregnancy cycle. In a previous population study by the present group, where the authors evaluated hospitalized pregnant and postpartum women with COVID-19, comparing 602 women with CVD with 2,960 who did not have CVD, we found that those with CVD were more symptomatic (*p <* 0.001), had a greater need for ICU admission (*p <* 0.001), ventilatory support (p = 0.004), and higher mortality rate (18.9 % vs. 13.5 %, *p <* 0.001). The risk of death was 32 % higher (OR = 1.32, 95 % CI 1.16–1.50), especially in the second (OR = 1.94, 95 % CI 1.43–2.63) and third (OR = 1.29, 95 % CI 1.04–1.60) trimesters, as well as in the puerperium period (OR = 1.27, 95 % CI 1.03–1.56).^6^

Several studies that evaluated the outcomes according to the temporal evolution of the pandemic revealed the possibility of more critical evolutions according to the predominant variant in circulation. Overall, they found a higher mortality associated with the second year of the pandemic, increased risk of ICU admission, and increased need for invasive ventilatory support in the general population.^31^^,^^32^

The second pandemic year in Brazil was marked by the increased prevalence of the Gamma variant, known to be more virulent. In this study, the authors observed worse clinical outcomes in pregnant and postpartum women with CVD in 2021, findings that can be attributed to exposure to a more lethal variant.

In a Brazilian population study that assessed the same period (February to December 2020 and January to May 2021) and evaluated 975,109 cases of patients hospitalized with COVID-19, there was no difference in the prevalence of CVD between the periods evaluated. In 2021, all groups evaluated (men, women outside the pregnancy-puerperal cycle, and pregnant and postpartum women) had more dyspnea (56 % vs. 69.4 %, OR = 1.78, 95 % CI 1.62‒1.96), desaturation (32 % vs. 54.3 %, OR = 2.52, 95 % CI 2.29‒2.78), OTI (10 % vs. 18.3 %, OR = 2.02, 95 % CI 1.78‒2.30) and mortality (7.5 % vs. 17.4 %, OR = 2.60, 95 % CI 2.28‒2.97).^33^ The assessment of this population, composed only of women with CVD, revealed similar outcomes, but with a higher prevalence of events in both periods under evaluation, with dyspnea affecting 80.4 % of patients in 2021 (OR = 1.93, 95 % CI 1.49‒2.48), desaturation in 69.2 % (OR = 1.91, 95 % CI 1.52–2.40) and higher mortality, with an increase from 15.5 % in 2020 to 25.6 % in 2021. These findings demonstrate the impact of CVD on the outcome of the obstetric population.

The data found corroborate other published studies that demonstrate that maternal morbidity and mortality were higher in the second year of the pandemic, despite the increase in the scientific community's knowledge about the disease.^33^^,^^34^

The strengths of this study include the use of a robust database in Brazil, in which there is compulsory notification of cases, and which has been in operation since 2009, with the pandemic caused by the H1N1 influenza virus. Since then, hospitalized cases of Severe Acute Respiratory Syndrome have been compulsorily notifiable in Brazil. The authors are therefore working with a consistent database, and the data compiled is reliable, allowing the evaluation of a significant number of hospitalized pregnant and postpartum women with CVD, that is, with a clinical picture of greater severity and with a high rate of laboratory confirmation of COVID-19 when compared to other published medical studies.

The documentation of a higher risk of severe involvement by SARs-CoV in the maternal, pregnant, and postpartum population is essential for the organization of resources and public policies. The authors are aware of the fragility of the public health system in Brazil, thus identifying women with CVD as a risk group can help implement differentiated health care, especially in relation to hospitalization and ventilatory support. Given the severity of the clinical evolution of CVD patients and pregnant women, understanding the evolution of the disease in these cases is essential so that the authors are prepared for other possible viral diseases or pandemics that may occur and that may target the cardiorespiratory system in the future, as occurred with COVID-19, and greater attention is given to this population aiming at reducing the risk of maternal death. In addition, this population is considered to be at higher risk from the outset and can receive specific health care, with greater vigilance, especially when the authors evaluate low adherence to vaccination and vaccine boosters.

The main limitations of the present study were the use of a national database, which depends on the adequate completion of information and, when evaluating only complete notifications to maintain reliability, the authors found a high number of losses. Furthermore, genomic isolation did not occur in the cases assessed, and the authors evaluated the strain with the highest population prevalence at the time of evaluation. It is worth mentioning that the authors selected the cases that were affected before the availability of the vaccine for the Brazilian obstetric population, and, therefore, the effects of immunization are not reflected in this study. Unfortunately, the form used for compulsory notification of Severe Acute Respiratory Syndrome cases in SIVEP-Gripe only contains information on the presence or absence of chronic cardiovascular disease as a personal history, without detailing the type of disease. This prevents us from evaluating the variants according to the CVD group.

## Conclusion

In Brazil, pregnant and postpartum women with CVDs in the Gamma variant phase have higher morbidity and mortality than those affected by the original variant of Coronavirus-19.

The higher prevalence of the Gamma variant in 2021 had a direct impact on patients with CVDs, with an increase in symptoms, ICU admissions, the need for invasive and non-invasive ventilatory support and, above all, maternal mortality, making the first 17 epidemiological weeks of 2021 the most lethal phase of the pandemic for the Brazilian obstetric population with CVDs.

## Supplementary materials

The following supporting information can be downloaded at: https://github.com/observatorioobstetrico/COVID19_CVD_2020vs2021, Fig. S1: title; Table S1: title; Video S1: title.

## Conflicts of interest

The authors declare no conflicts of interest.
